# Forkhead Box O1 Is Present in Quiescent Pituitary Cells during Development and Is Increased in the Absence of *p27^Kip1^*

**DOI:** 10.1371/journal.pone.0052136

**Published:** 2012-12-14

**Authors:** Sreeparna Majumdar, Corrie L. Farris, Brock E. Kabat, Deborah O. Jung, Buffy S. Ellsworth

**Affiliations:** Department of Physiology, Southern Illinois University, Carbondale, Illinois, United States of America; St. Vincent's Institute, Australia

## Abstract

Congenital pituitary hormone deficiencies have been reported in approximately one in 4,000 live births, however studies reporting mutations in some widely studied transcription factors account for only a fraction of congenital hormone deficiencies in humans. Anterior pituitary hormones are required for development and function of several glands including gonads, adrenals, and thyroid. In order to identify additional factors that contribute to human congenital hormone deficiencies, we are investigating the forkhead transcription factor, FOXO1, which has been implicated in development of several organs including ovary, testis, and brain. We find that FOXO1 is present in the nuclei of non-dividing pituitary cells during embryonic development, consistent with a role in limiting proliferation and/or promoting differentiation. FOXO1 is present in a subset of differentiated cells at e18.5 and in adult with highest level of expression in somatotrope cells. We detected FOXO1 in p27^Kip1^-positive cells at e14.5. In the absence of *p27^Kip1^* the number of pituitary cells containing FOXO1 is significantly increased at e14.5 suggesting that a feedback loop regulates the interplay between FOXO1 and *p27^Kip1^*.

## Introduction

Pituitary organogenesis begins on embryonic day 7.5 in mice [1], [2]. By e8.5 Rathke’s pouch begins to form from the oral ectoderm. Initially, Rathke’s pouch is a rudimentary structure that extends in a caudal direction. Apoptosis occurs at approximately e10.5 to separate Rathke’s pouch from the oral ectoderm that will form the mouth [1], [3], [4]. The cells located around the lumen of Rathke’s pouch are undifferentiated and actively proliferating. These cells migrate ventrally to form the anterior lobe. It is here they differentiate into differentiated cell types. Corticotrope cells secrete adrenocorticotropic hormone (ACTH), which regulates adrenal function. Somatotrope cells secrete growth hormone (GH), which stimulates long bone growth. Lactotrope cells secrete prolactin (PRL), which is important for mammary gland function. Thyrotrope cells secrete thyroid-stimulating hormone (TSH), which regulates thyroid gland function. Finally, Gonadotrope cells secrete luteinizing hormone (LH) and follicle-stimulating hormone (FSH), which are essential for normal reproductive function [1], [2]. These hormone secreting cell types differentiate in a temporal order [5].

Various mouse models have identified several transcription factors that are necessary for pituitary development and result in combined pituitary hormone deficiency in humans [1], [6]. Lesions in the transcription factor genes, *LHX3, LHX4, RPX, PROP1,* and *PIT1* contribute to 13% of combined pituitary hormone deficiencies in humans [7]. As mutations in these transcription factors account for only a fraction of congenital hormone deficiencies, it is necessary to identify other transcription factors that are important in pituitary development in order to expand the molecular diagnoses available for pituitary hormone deficiencies.

FOXO1 is a forkhead transcription factor that inhibits proliferation and cell migration and regulates cell differentiation in a number of organs [8], [9], [10], [11], [12]. For example, FOXO1 regulates epithelial cell migration in blood vessels and loss of FOXO1 causes increased vessel sprouting [12]. Mouse knockout models for *Foxo1* (*Foxo1^−/−^*) result in embryonic lethality at e10.5 due to lack of vascularization [13]. Loss of FOXO1 specifically in muscle results in increased formation of fast-twitch muscle fibers and decreased formation of slow-twitch fibers [10], suggesting that FOXO1 is important for cell fate determination. During muscle fiber differentiation FOXO1 works with the NOTCH receptor to inhibit myoblast differentiation. FOXO1 represses expression of *Pdx* in mature pancreatic β-cells. PDX is also important for pancreas morphogenesis and is expressed in the same cells as FOXO1, but in different subcellular locations, suggesting that FOXO1 inhibition of *Pdx* expression may be required for normal pancreas morphogenesis [9], [14]. Much remains to be learned about the identity of factors that determine pituitary cell fate. Because the signaling molecule, NOTCH, is known to play a role in pituitary development and can interact with FOXO1 [10], [15], [16], [17], [18], [19], we examined the expression of FOXO1 in the presence and absence of NOTCH signaling targets. We find that FOXO1 is present in quiescent pituitary cells during development and its normal expression pattern is altered in the absence of the cell cycle regulator, p27^Kip1^.

**Figure 1:**
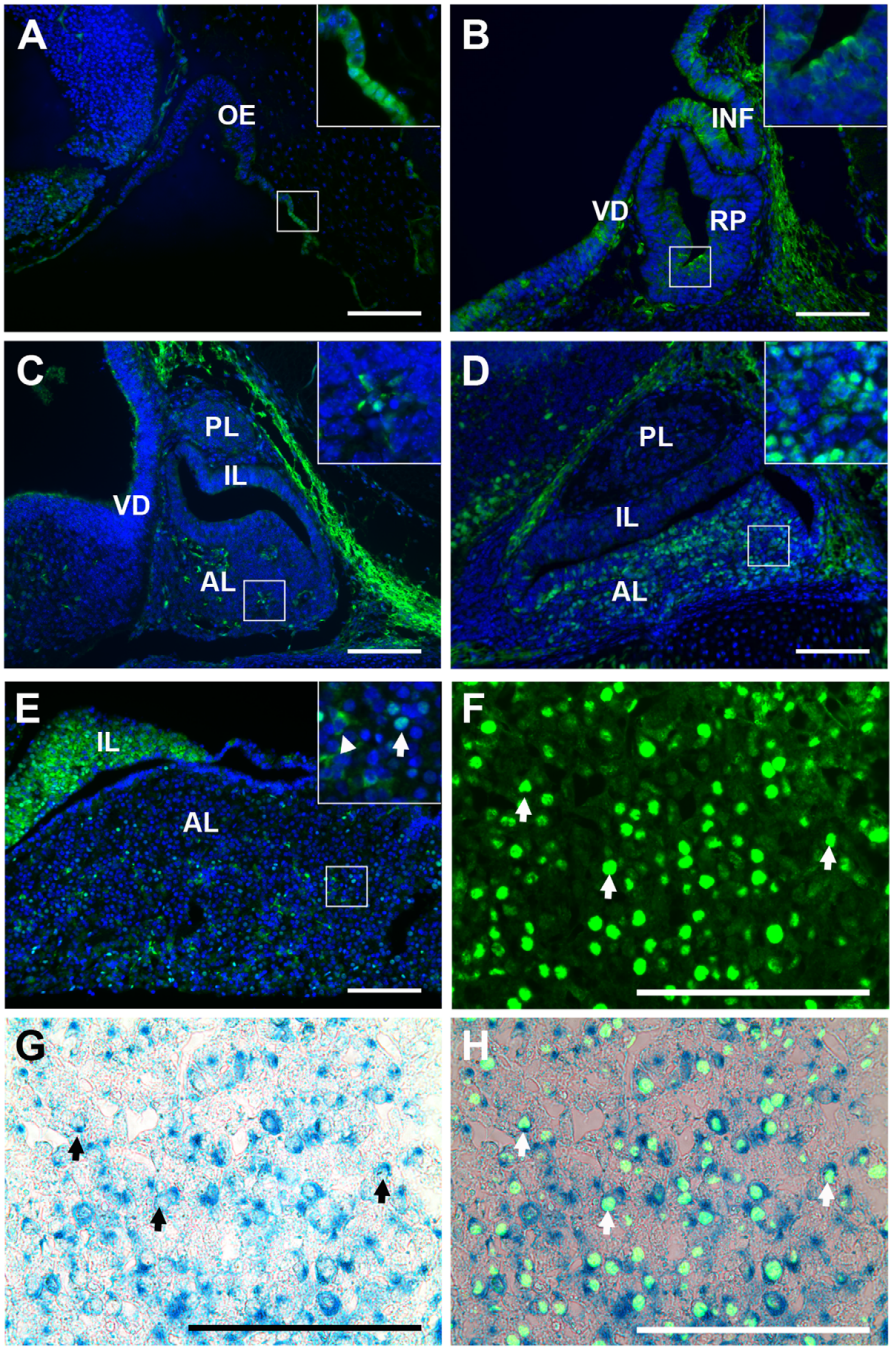
FOXO1 is present in the nuclei of pituitary cells at an increasing frequency as development progresses. Immunohistochemistry for FOXO1 (green) was performed on midsagittal pituitary sections. (A) FOXO1 is present in the developing pituitary by e10.5. Nuclear FOXO1 is apparent where the invaginating Rathke’s pouch is joined to the oral ectoderm that will form the mouth (see inset). (B) By e12.5 FOXO1 is present almost entirely in the cytoplasm of pituitary cells. (C) A few pituitary cells contain nuclear FOXO1 at e14.5. (D) At e18.5 the developing pituitary contains a mostly nuclear FOXO1. (E) In adults, FOXO1 is present in the anterior and intermediate lobes of the pituitary gland, but not in the posterior lobe (data not shown). In the adult pituitary FOXO1 is primarily nuclear (inset, arrow). Some cytoplasmic FOXO1 (inset, arrow head) is also present. (F) Immunohistochemistry for FOXO1. (G) Beta-galactosidase staining of pituitary from *Foxo1^+/LacZ^* mice identifies cells in which the endogenous *Foxo1* promoter is active. (H) An overlay of immunohistochemical staining for FOXO1 (green) and β-galactosidase staining of pituitary from *Foxo1^+/LacZ^* mice (blue). (F–H) Arrows highlight examples of co-localized cells. Pictures are taken at 200X (A–E) or 630X (F–H). Insets are magnified 600X. Scale bars represent 100 µm. All cell nuclei were marked with DAPI (A–E, blue). Oral ectoderm (OE), infundibulum (INF), ventral diencephalon (VD), Rathke’s pouch (RP), posterior lobe (PL), intermediate lobe (IL), anterior lobe (AL).

## Materials and Methods

### Ethics Statement

This study was carried out in strict accordance with the recommendations in the Guide for the Care and Use of Laboratory Animals of the National Institutes of Health. The protocol was approved by the Southern Illinois University Animal Care and Use Committee (Protocol Number: 10–020).

### Mice

C57BL/6J mice were purchased from Jackson Laboratories. Mice were maintained in a 12-hour dark-light cycle and fed Purina Mills Formulab diet 5008 ad libitum. Embryos were obtained from an intercross of C57BL/6J mice. The morning the copulatory plug was detected was designated to be e0.5. The targeted allele of *Foxo1^+/LacZ^* mice was engineered by replacing the coding region of exon 1 and approximately 3 kb of intron 1 with a β-geo cassette as described previously [20]. Tissues from *Hes1* [16], [21], [22], Ames dwarf [19], [23], [24], [25], [26], and *p27^Kip1^* (B6.129S40-*Cdkn1b^tm1Mlf^*/J) [27], [28] embryos were generously provided by Lori T. Raetzman at the University of Illinois.

### Histology and Immunohistochemistry

Embryos were dissected and fixed in 4% paraformaldehyde in phosphate buffered saline (PBS) (pH 7.2) for 45 min to 24 h (depending on stage of development). All samples were washed in PBS, dehydrated in a graded series of ethanol, and embedded in paraffin. Sections (5 µm) were deparaffinized in xylene and rehydrated through a series of graded ethanol washes before immunohistochemistry was performed.

To visualize FOXO1, tissue sections were deparaffinized and rehydrated as described above, and 1.5% peroxide in water was used to remove endogenous peroxidases. After epitopes were unmasked by boiling in 10 mM citric acid for 10 min, tissue sections were blocked with blocking reagent from the Tyramide Signal Amplification kit (PerkinElmer). Sections were incubated overnight at 4°C with antibodies that specifically recognize FOXO1 (Cell Signaling, 1∶50). Tissue sections were incubated with anti-rabbit secondary (Jackson ImmunoResearch Laboratories, Inc) for 30 min at room temperature. Next, sections were incubated sequentially with streptavidin-horseradish peroxidase and fluorescein from the Tyramide Signal Amplification kit (PerkinElmer). Following a 5 min incubation with water, sections were counterstained with 4′,6-diamidino-2-phenylindole (DAPI) (167 nM, Molecular Probes).

**Figure 2:**
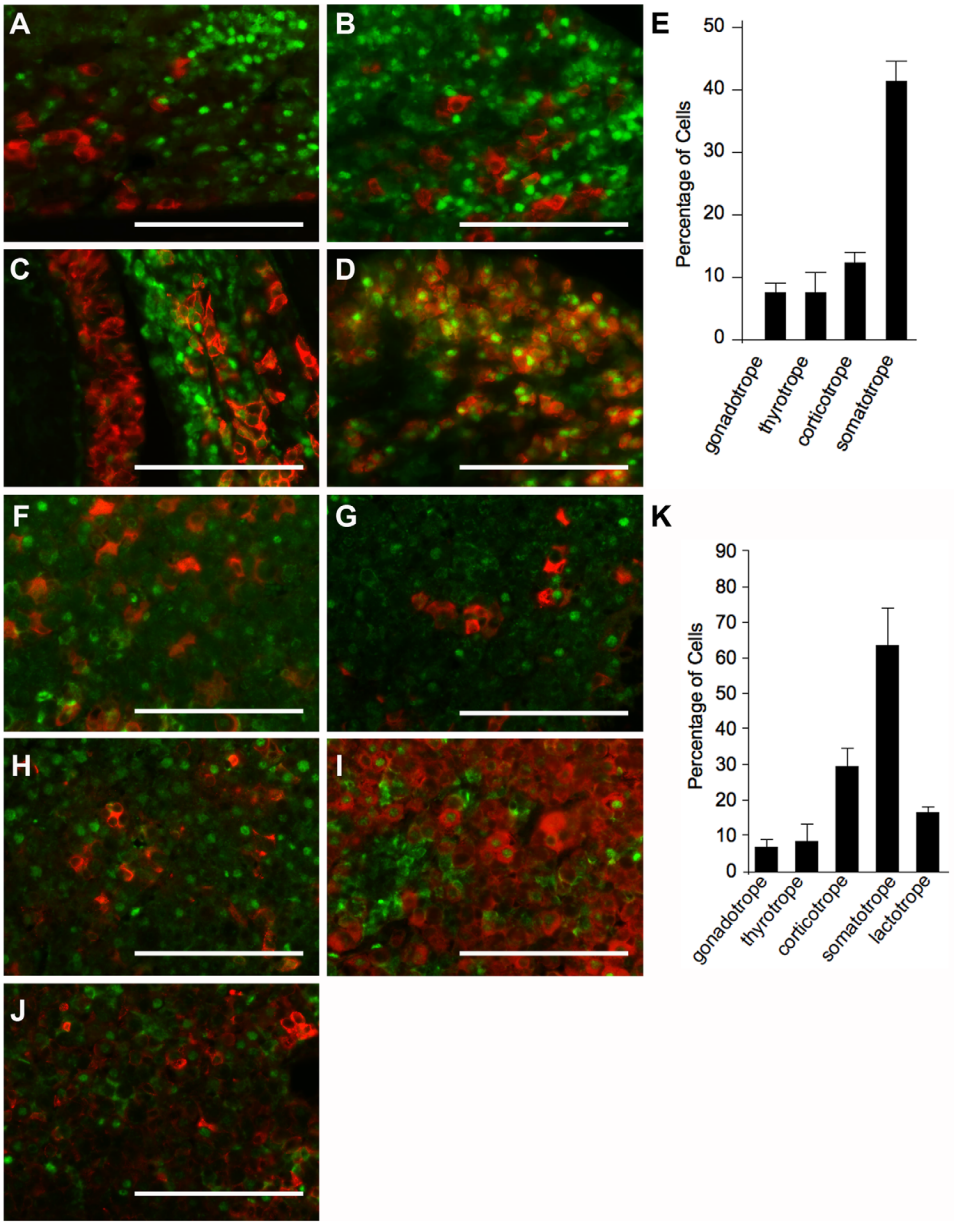
Approximately half of somatotrope cells contain nuclear FOXO1. Hormone co-localizations were performed on embryonic pituitary tissue at e18.5 (A–E) and in adults (F–K). FOXO1 (green) and LH (A, F), TSH (B, G), ACTH (C, H), GH (D, I), or PRL (J) were labeled by immunohistochemistry. Hormones are shown in red. The total number of hormone-positive cells and the number of hormone cells containing nuclear FOXO1 were counted manually. Pictures were taken at 630X. Scale bars represent 100 µm. (E) In the e18.5 embryonic pituitary, nuclear FOXO1 is present in 8% of gonadotrope cells, 8% of thyrotrope cells, 13% of corticotrope cells, and 42% of somatotrope cells. (K) In the adult pituitary gland, nuclear FOXO1 is present in 7% of gonadotrope cells, 9% of thyrotrope cells, 30% of corticotrope cells, 63% of somatotrope cells, and 15% of lactotrope cells. Graphs represents mean ± SEM.

Co-localization of FOXO1 with hormones was performed as described above except tissue sections were boiled in 10 mM citric acid for only 5 min. To visualize pituitary hormones, tissue sections were incubated with antibodies against growth hormone (GH; 1∶10,000; National Hormone National Hormone and Peptide Program (NHPP)), pro-opiomelanocortin (POMC; 1∶500; NHPP), thyroid-stimulating hormone beta subunit (TSHB; 1∶2000, NHPP), or luteinizing hormone beta subunit (LHB; 1∶500, NHPP) for 1 hour at room temperature and then the appropriate secondary antibodies: anti-rabbit-TRITC (1∶100, Jackson ImmunoResearch) or anti-guinea pig-FITC (1∶100, Jackson ImmunoResearch).

To detect cell proliferation in embryonic pituitaries, pregnant mice were given an intraperitoneal injection of bromodeoxyuridine (BrdU) at 100 µg/g body weight 2 h before the embryos were collected [29].

Co-localization studies with FOXO1 and BrdU were performed as follows. Tissue sections were deparaffinized and rehydrated as described above, and 1.5% peroxide in water was used to block endogenous peroxidases. Epitopes were unmasked by boiling in 10 mM citric acid for 10 min and immunohistochemistry for FOXO1 was performed as described above. After incubation with fluorescein, tissue sections were blocked using the Mouse on Mouse (M.O.M.) kit (Vector Laboratories) according to manufacturer's directions and then blocked with biotin solution and streptavidin solution (Vector Laboratories) according to manufacturer’s directions. Tissue sections were incubated overnight at 4°C with antibodies for BrdU (Invitrogen, clone ZBU30, 1∶100) followed by incubation with anti-mouse secondary (M.O.M. kit, Vector Laboratories) 30 min at room temperature. Next, sections were incubated sequentially with streptavidin-horseradish peroxidase and fluorescein from the Tyramide Signal Amplification kit (PerkinElmer).

Co-localization studies with FOXO1 and p27 or p57 were performed as described for BrdU except after immunohistochemistry for FOXO1, tissue sections were incubated overnight with antibodies for either p27^Kip1^ (1∶30, BD Pharmingen) or p57^Kip2^ (1∶50, Santa Cruz Biotechnology) followed by incubation with biotinylated anti-mouse or biotinylated anti-rabbit secondary antibodies, respectively.

Digital images of pituitary sections were captured with a Leica DM 5000B fluorescent microscope and Retiga 2000R digital camera. FITC, TRITC, and DAPI pictures were merged using Adobe Photoshop CS3.

**Figure 3:**
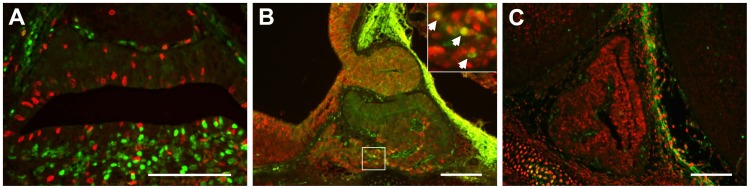
FOXO1 is present in quiescent pituitary cells during development. (A) FOXO1 (green) is not present in actively dividing cells labeled with BrdU (red) at e16.5. Picture was taken at 400X. (B) FOXO1 (green) is present in a subset of p27-positive cells (red) at e14.5. Picture was taken at 200X. (C) FOXO1 (green) is not present in cells exiting the cell cycle that are expressing p57 (red) at e14.5. Picture was taken at 200X. Scale bars represent 100 µm.

Cells were counted manually. One-two mid-sagittal sections per sample were counted. Photographs were taken at 630X of one - two different fields per slide for FOXO1/hormone co-localization or at 200X of one field per slide for the presence of FOXO1 in *Hes1, Prop1,* and *p27* mutants. Three - five different individuals were examined for each group. Only cells containing nuclear FOXO1 were counted. Nuclear FOXO1 was defined as cells in which FITC co-localized with DAPI, a nuclear marker.

### β-galactosidase Staining

Pituitary glands from adult *Foxo1^+/LacZ^* mice were stained whole mount for β-galactosidase as follows. Embryos and adult pituitaries were fixed in 4% formaldehyde for 1 hour, rinsed in PBS and stained overnight in β-galactosidase staining solution (5 mM potassium ferricyanide, 5 mM potassium ferrocyanide, 1 mg/mL X-gal in 1X PBS). After a series of graded ethanol washes, samples were embedded in paraffin and sectioned (5 µm). Immunohistochemistry for FOXO1 was performed on these tissue sections as described above.

### Statistical Analysis

All results are expressed as mean ± SEM. Data were analyzed by Student’s t-test using Microsoft Excel. P-values less than 0.05 are considered significant (*).

**Figure 4:**
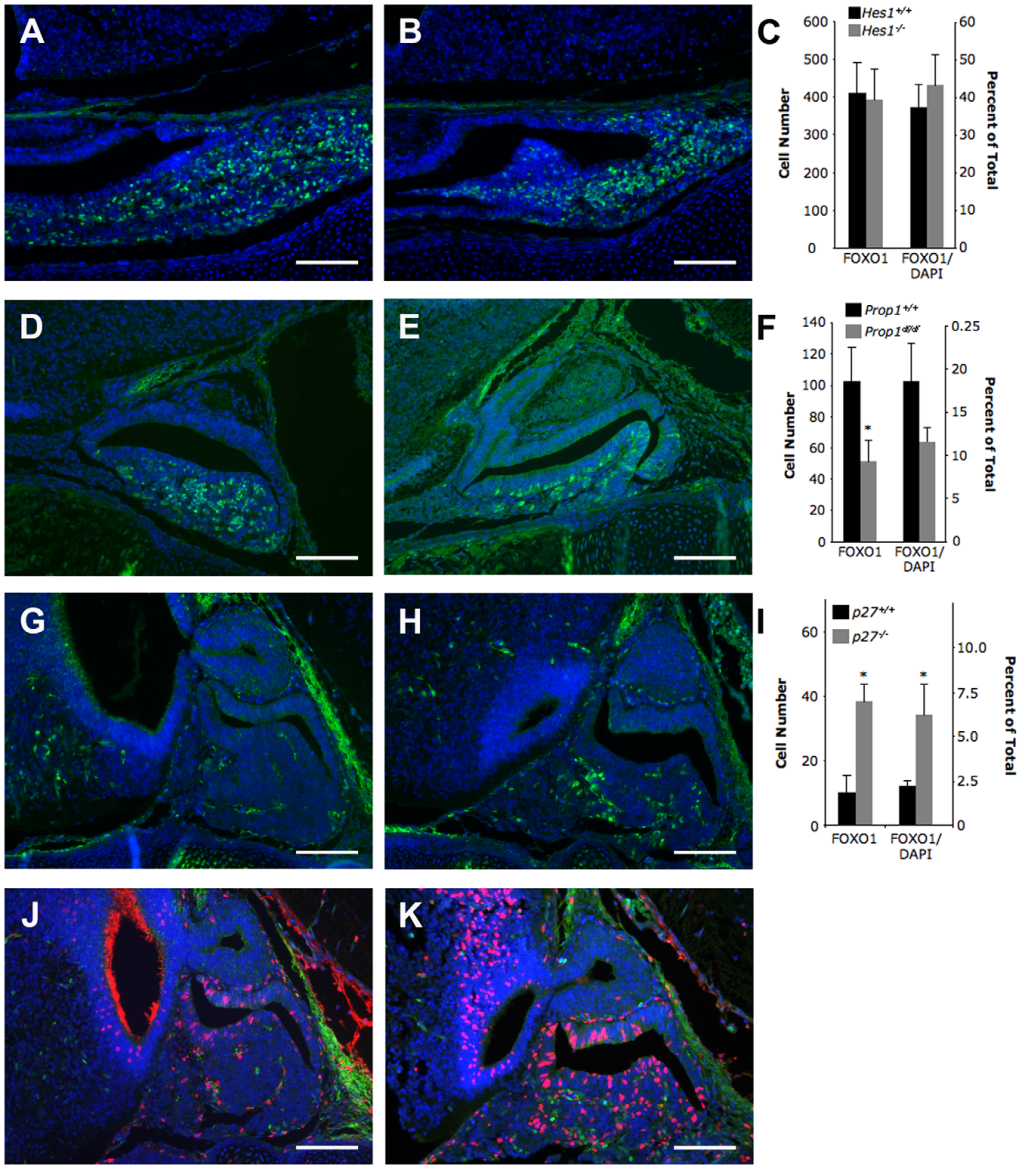
The number of FOXO1-positive pituitary cells is increased in the absence of *p27^Kip1^*. FOXO1 (green) was analyzed in mouse models that lack several different pituitary transcription factors. The number of FOXO1-positive pituitary cells and the total number (DAPI-positive) of pituitary cells were counted manually. Graphs represent mean ± SEM. The left y-axis shows the total number of FOXO1-positive pituitary cells. The right y-axis represents the number of FOXO1-positive pituitary cells as a percentage of the total number of pituitary cells. (A–C) FOXO1 is not different between *Hes1* null embryos and wild type littermates at e16.5. (D–F) The number of FOXO1-positive cells is significantly reduced in *Prop1^df/df^* mice as compared to wild type littermates at e16.5. However, when the reduction in the total number of pituitary cells is considered the difference is no longer significant. (G–I) The number of cells containing FOXO1 is increased approximately 3-fold in *p27^Kip1^* null mice at e14.5. The percentage of pituitary cells containing FOXO1 is increased by approximately 2.5-fold in these mutants. (J–K) FOXO1 (green) does not co-localize with BrdU (red) suggesting that FOXO1-positive cells remain quiescent in the absence of *p27^Kip1^*. Student’s t-test was performed to determine significance (*P<0.05). Sample size is three for these studies. Pictures are taken at 200X. Scale bars represent 100 µm.

## Results

### FOXO1 Expression During Pituitary Development

During pituitary development there are distinctive locations and timing for the emergence of proliferating progenitor cells and the terminal differentiation of individualized cell types. To define the spatial and temporal patterns of FOXO1 protein and to implicate its potential role in these processes, immunohistochemistry was performed with antibodies specific for FOXO1. Nuclear FOXO1 protein is observed at e10.5 in the region where the developing Rathke’s pouch is adjoined to the portion of the oral ectoderm that will form the oral cavity (Fig. 1A). At e12.5, a period of rapid proliferation, FOXO1 is excluded from the nucleus of pituitary cells (Fig. 1B). A small fraction of pituitary cells contain nuclear FOXO1 at e14.5 (Fig. 1C). By e16.5 an increased number of cells in the anterior pituitary contain nuclear FOXO1 (Fig. 1D, data not shown). FOXO1 is not present in the intermediate lobe during development (Fig. 1A–D). The percentage of pituitary cells containing FOXO1 protein in the nucleus increases throughout development as proliferation decreases, consistent with a role for inhibiting cell proliferation and/or regulating cell differentiation. Consistent with our observations of the pituitary early in development, FOXO1 is cytoplasmic in the ventral diencephalon and infundibulum at e10.5 and e12.5 (data not shown and Fig. 1B). While the infundibulum will form the posterior lobe, FOXO1 is not present in the posterior lobe after e14.5 (Fig. 1C–D and data not shown). Although FOXO1 is becoming nuclear in the anterior lobe of the pituitary gland at e14.5, no nuclear FOXO1 is present in the ventral diencephalon or the infundibulum. In fact, FOXO1 is barely detectable in these regions by e14.5 (Fig. 1C, and data not shown).

In the adult FOXO1 is present in the intermediate lobe and the anterior lobe, but not in the posterior lobe (Fig. 1E and data not shown). In the anterior lobe FOXO1 is mostly nuclear, but cytoplasmic FOXO1 is also present (Fig. 1E inset). To validate immunohistochemical staining of FOXO1, β-galactosidase staining was performed on pituitary from *Foxo1^+/LacZ^* mice [20]. In *Foxo1^+/LacZ^* mice, β-galactosidase expression is regulated by the *Foxo1* promoter, thus blue cells correspond with *Foxo1* expression. Immunohistochemistry for FOXO1 coincides with β-galactosidase expression (Fig. 1 F–H).

### FOXO1 is Present in a Subset of Hormone-Producing Pituitary Cells

The anterior pituitary contains at least five different hormone-secreting cell types, each with its own expression profile. Knowing which cell types normally contain FOXO1 protein is important for understanding its contribution to pituitary gland development. To determine which cell types contain FOXO1, the hormones that mark each pituitary cell type were labeled together with FOXO1 in embryonic pituitary sections at e18.5. Lactotrope cells were not examined at this age because PRL is not consistently detectable by immunohistochemistry before birth. FOXO1 is present in the nuclei of approximately 8% of LH-positive cells (Fig. 2A), 8% of TSH-positive cells (Fig. 2B), 13% of ACTH-positive cells (Fig. 2C), and 42% of GH-positive cells (Fig. 2D). The fact that FOXO1 is not present in 100% of any cell type (Fig. 2E) suggests that FOXO1 is not required for expression of *Lhb*, *Tshb*, *Pomc*, or *Gh*. It is intriguing to postulate what may be different about this subset of hormone-producing cells that is marked by FOXO1. FOXO1 is present in a similar distribution in the adult pituitary gland (Fig. 2F–K). Co-localizations between FOXO1 and PRL were performed on adult pituitary and approximately 15% of adult lactotrope cells contain nuclear FOXO1 (Fig. 2J–K).

### FOXO1 is Present in Quiescent Anterior Pituitary Cells

Developmental expression analysis of FOXO1 in the pituitary gland shows that detection of FOXO1 protein in pituitary cell nuclei increases throughout development as pituitary cell proliferation decreases, suggesting that FOXO1 is present in the nuclei of quiescent cells (Fig. 1). To determine if FOXO1 protein is, in fact, present in the nuclei of quiescent cells, pregnant mice were injected with the thymidine analog, bromodeoxyuridine (BrdU, 100 µg/g body weight), 2 hours before tissue collection to label dividing cells in developing embryos. Embryonic mouse pituitaries were analyzed at e16.5, a time point when FOXO1 exhibits nuclear expression at significant levels in the pituitary gland. Tissues were fixed, embedded, and immunohistochemistry was completed to co-label FOXO1 and BrdU. FOXO1 is not present in nuclei of proliferating pituitary cells at e16.5 (Fig. 3A) or at e14.5 (data not shown). Two cyclin-dependent kinase inhibitors, p27^Kip1^ and p57^Kip2^ have important roles during pituitary development [30]. The role of p27^Kip1^ is to prevent re-entry of differentiated cells into the cell cycle [30]. FOXO1 inhibits proliferation in muscle by stimulating expression of *p27^Kip1^* [31]. A few pituitary cells contain nuclear FOXO1 at e14.5 and these co-localize with a fraction of p27^kip1^-positive pituitary cells (Fig. 3B). The proposed role of p57 during pituitary development is to promote cell cycle exit of pituitary progenitor cells [32]. We find that FOXO1 is not present in p57-positive pituitary cells (Fig. 3C). Together, these data are consistent with a role for FOXO1 of inhibiting cell proliferation and/or promoting cell differentiation.

### Interactions between FOXO1 and Other Pituitary Genes

Interactions between transcription factors are required for normal pituitary development. FOXO1 is important for inhibiting cell cycle progression in many tissues including myocardium, ovarian granulosa, and pancreatic β-cells [9], [33], [34]. To understand how FOXO1 interacts with known pituitary transcription factors and regulators of cell cycle progression to control pituitary development we analyzed FOXO1 protein in several different mouse mutants. The NOTCH1 target, HES1, is a transcriptional repressor of genes coding for cell cycle inhibitors [35], [36], [37]. Our analyses reveal that neither the total number of pituitary cells containing FOXO1 nor the percentage of pituitary cells containing FOXO1 is different between wild type embryos (Fig. 4A) and *Hes1* null littermates (Fig. 4B) at e16.5 (Fig. 4C), suggesting that FOXO1 levels are not affected either directly or indirectly by HES1. Ames dwarf mice have a null mutation in the *Prop1* gene [23], [25]. Mice and humans with loss of PROP1 function have very reduced numbers of cells in the PIT1 lineage, thyrotrope, somatotrope, and thyrotrope cells [2], [38]. When comparing wild type embryos (Fig. 4D) and *Prop1^df/df^* littermates (Fig. 4E), we find a reduced number of FOXO1-positive cells in *Prop1^df/df^* mice (Fig. 4F). However, when the reduction in the total number of anterior pituitary cells is considered, the difference in FOXO1-containing cells is no longer statistically significant (Fig. 4F), suggesting that FOXO1 production is not affected by PROP1. The cyclin-dependent kinase inhibitor, p27^Kip1^, is important for maintaining pituitary cells in an undifferentiated state [30]. FOXO1 is present in the nuclei of very few pituitary cells in wild type embryos at e14.5 (Fig. 4G). We find that FOXO1 is present in an increased number of cell nuclei in littermates lacking p27^Kip1^ (Fig. 4H, I). These data suggest that FOXO1 is involved in an interesting feedback mechanism to compensate for the absence of p27^Kip1^. Bilodeau et al. found that loss of p27^Kip1^ caused some POMC-containing cells to proliferate at e14.5 [30]. Normally, FOXO1 is present only in non-proliferating cells (Fig. 3A). To determine if loss of p27^Kip1^ caused proliferation of FOXO1-containing cells we performed co-localization studies with FOXO1 and BrdU on wild type embryos (Fig. 4J) and *p27^Kip1^* null littermates (Fig. 4K). No co-localization between FOXO1 and BrdU was detected demonstrating that loss of p27 does not cause proliferation of FOXO1-positive cells (Fig. 4J–K).

## Discussion

In the present study, the spatial and temporal expression of FOXO1 was investigated in anterior pituitary cells of mouse pituitary at different time points. FOXO1 immunopositive cells were observed in the anterior pituitary cells throughout pituitary development, appearing by embryonic day 10.5. Immunohistochemical data demonstrating a significant increase in nuclear FOXO1 from e14.5 to e18.5, a period of decreasing cell proliferation and increasing cell differentiation, suggested that FOXO1 might be important for regulation of genes that regulate cell cycle progression, differentiation, and/or function. A similar *Foxo1* expression pattern has been reported in mouse brain where in situ hybridization was used to demonstrate that onset of expression occurred around e14.5 and became intensified by e18.5, however subcellular localization of FOXO1 was not investigated [39].

The ventral diencephalon and infundibulum secrete many signaling factors that are important for directing pituitary development [2], [40], [41], [42]. Early in development, FOXO1 is present in the cytoplasm of pituitary tissues and the neighboring tissues, ventral diencephalon and infundibulum. Thus, early in development, FOXO1 may be regulated in a similar manner in both the pituitary and ventral diencephalon. However, after e14.5, FOXO1 is no longer detectable in the ventral diencephalon. Because nuclear FOXO1 is not present in these tissues it is unlikely that FOXO1’s presence in the ventral diencephalon or infundibulum affects pituitary development.

Hypothalamic releasing hormones and dopamine are critical for regulating pituitary function [2], [43]. FOXO1 is expressed in POMC and AgRP neurons of the hypothalamus [44]. Whether hypothalamic FOXO1 also localizes to releasing hormone or dopaminergic neurons has not been investigated. Further studies will be required to determine if hypothalamic FOXO1 affects pituitary function.

These studies reveal that FOXO1 is present primarily in the nucleus after e16.5 and continuing throughout adulthood. Also, FOXO1 is present in approximately 50% of somatotrope cells and less than 10% of gondaotrope cells. This is different from what was observed by Arriola et al., who found that FOXO1 was almost entirely cytoplasmic in adult mouse pituitary and present in nearly all gonadotrope and thyrotrope cells [45]. One possibility for these differences is the use of different antibodies. However, we still observe nuclear localization of FOXO1 with the antibody used by Arriola et al. (Santa Cruz, H-128, data not shown) suggesting that this is not the cause of the discrepancy. Another possibility for the differences observed is that the mice may be on different diets, light-dark cycles, or have different genetic backgrounds. This is a potentially interesting phenomenon and needs to be investigated further.

Consistent with the spatial and temporal pattern of FOXO1 protein in pituitary during development, co-localization studies with BrdU demonstrated that FOXO1 is not present in actively dividing cells. Along these lines, we sought to determine if FOXO1 is present in pituitary cells that are exiting the cell cycle, as marked by p57, or are differentiating, as marked by p27 and the presence of hormone. We found that FOXO1 is not present in pituitary cells that are exiting the cell cycle, but rather is present in a subset of differentiated cells. This suggests that FOXO1 may be important either for differentiation or maintaining differentiated cells in a non-proliferative state. FOXO1 inhibits proliferation in blood B-cells and pancreatic endocrine cells and is required for B-cell differentiation [14], [46], [47]. In myocytes, loss of FOXO1 increases differentiation of fast-twitch muscle fibers and decreases differentiation of slow-twitch fibers [10].

During muscle differentiation FOXO1 cooperates with NOTCH to activate NOTCH target genes, including *Hes1* [10]. This study suggests that this interaction may integrate environmental cues via NOTCH and metabolic cues via FOXO1. If a similar scenario may be present in the developing pituitary where FOXO1 and NOTCH interact to activate *Hes1* we would expect *Hes1* to be downstream of FOXO1 and would not expect to see a change in FOXO1 in the absence of *Hes1*. This is consistent with our findings.

The number of pituitary cells containing FOXO1 is increased in the absence of *p27*. During cell cycle progression retinoblastoma (RB) sequesters the transcription factor E2F effectively preventing it from stimulating expression of cyclin E and other S-phase promoting genes [48]. RB can be phosphorylated by a complex containing cyclin-dependent kinase 2 (Cdk2) and cyclin E resulting in release of E2F and cell cycle progression [49]. Inactivation of Cdk2 by p27 maintains the interaction between RB and E2F and inhibits cell cycle progression [49]. Interestingly, expression of the *Foxo1* gene is stimulated by E2F [50]. Thus, the increase in the number of FOXO1-positive cells observed in *p27* null embryos is possibly due to an increase in free E2F caused by an increase in RB phosphorylation in the absence of p27.

FOXO1 is present in a subset of differentiated cell types with the largest percentage present in somatotrope cells. This suggests that FOXO1 may have a functional role in this cell type. FOXO1 is tightly regulated post-translationally by insulin and insulin-like growth factor (IGF1) and is important for integrating these metabolic signals in hepatocytes, hypothalamic POMC neurons, and adipocytes [9]. Growth hormone production is regulated by IGF1 and insulin [51]. Insulin also affects gonadotrope function by enhancing gonadotropin-releasing hormone stimulation of LH expression and secretion [52], [53], [54], [55]. FOXO1 is highly regulated by insulin signaling [56]. Brothers et al. demonstrated that loss of the insulin receptor reduces LH levels in obese female mice [52]. It is intriguing to postulate that FOXO1 may serve as an intermediate for communication between these metabolic signals and pituitary function. In order to determine whether FOXO1 is important for sensing metabolic signals for the pituitary gland, more studies will be necessary. Studies of mice lacking *Foxo1* specifically in the pituitary gland will be particularly interesting.
